# Acute EBV-Associated Systemic Inflammatory Syndrome Presenting as PUO With FDG-Avid Generalized Lymphadenopathy, Autoimmune Serological Positivity and Myopericarditis Mimicking Lymphoma and Connective Tissue Disease: A Diagnostic Challenge

**DOI:** 10.7759/cureus.113665

**Published:** 2026-07-30

**Authors:** Abdul Haleem Abbasi, Mridhu Job, Fahmida Faiza, Ameena Rahman, Hina Kamal

**Affiliations:** 1 General Internal Medicine, Broomfield Hospital, Chelmsford, GBR; 2 General Medicine, Broomfield Hospital, Chelmsford, GBR

**Keywords:** autoimmune-like, ebv positive, myopericarditis, pyrexia of unknown origin (puo), reactive lymphadenopathy

## Abstract

Pyrexia of unknown origin (PUO) remains a diagnostic challenge, particularly when associated with lymphadenopathy, multisystem involvement and positive autoimmune serology. Distinguishing between infectious, inflammatory and malignant causes is often difficult, especially when advanced imaging demonstrates findings suggestive of lymphoma.

A previously healthy man in his late 40s presented with a 10-day history of intermittent fever, myalgia, joint stiffness and pleuritic chest pain. During admission, he developed photophobia, mild neck stiffness, progressive upper and lower limb pain and weakness resulting in impaired mobility. Investigations demonstrated marked systemic inflammation with a C-reactive protein (CRP) of 235 mg/L and an erythrocyte sedimentation rate (ESR) of 105 mm/hr despite a normal white cell count (WCC). Extensive microbiological investigations including blood cultures, cerebrospinal fluid analysis, bacterial and fungal molecular testing, tuberculosis screening and viral polymerase chain reaction (PCR) testing were negative apart from detectable Epstein-Barr virus (EBV) DNA and positive EBV IgM serology. Autoimmune screening revealed positive ANA, anti-Ro52, anti-Ro60 and anti-La antibodies with low complement C4. Cardiac magnetic resonance imaging (MRI) confirmed acute myopericarditis. Positron emission tomography*-*computed tomography (PET-CT) demonstrated fluorodeoxyglucose (FDG)-avid supra- and infradiaphragmatic lymphadenopathy with splenic involvement highly suspicious for lymphoma. However, excisional cervical lymph node biopsy demonstrated reactive lymphoid hyperplasia without evidence of malignancy. Clinical improvement began approximately during the second week after admission and occurred without immunosuppressive therapy.

## Introduction

Pyrexia of unknown origin (PUO) remains one of the most challenging presentations encountered in general internal medicine, with a broad differential diagnosis that continues to pose diagnostic difficulty [1]. Despite advances in molecular diagnostics and imaging techniques, the differential diagnosis includes infections, autoimmune disorders, inflammatory diseases and malignancy.

Lymphoma is a key diagnostic consideration in PUO, particularly when associated with lymphadenopathy and splenomegaly [2].

Epstein-Barr virus (EBV) infects more than 90% of adults worldwide and is best known as the cause of infectious mononucleosis [3-5]. However, EBV infection may present with a wide spectrum of clinical manifestations beyond the classical triad of fever, pharyngitis and lymphadenopathy [3,4]. EBV infection can also induce transient autoantibody formation through immune dysregulation and molecular mimicry [6], which may resemble systemic autoimmune disease. It has been associated with hepatitis, myocarditis, pericarditis and polyclonal immune activation [3,4,7]. In addition, EBV-associated lymphadenopathy may show significant fluorodeoxyglucose (FDG) uptake on positron emission tomography (PET) imaging, making differentiation from lymphoma challenging [2].

We report a previously healthy man presenting with PUO, FDG-avid generalized lymphadenopathy, splenic involvement, autoimmune serological positivity, inflammatory musculoskeletal features and cardiac magnetic resonance imaging (MRI)-confirmed myopericarditis.

## Case presentation

A previously fit and well gentleman in his late 40s presented to the emergency department with a 10-day history of intermittent fever, generalized myalgia, joint stiffness and intermittent pleuritic chest pain. He lived on a boat with his partner and had no significant past medical history. He was a non-smoker, took no regular medications and remained fully independent in all activities of daily living. There was no recent travel history, exposure to farm animals, insect bites or known infectious contacts. On presentation, he described recurrent fevers reaching 39°C associated with profound malaise, diffuse muscle aches and generalized stiffness. Clinical examination did not identify a focal source of infection. Cardiovascular and respiratory examinations were unremarkable apart from pleuritic chest discomfort. There was no rash, synovitis, oral or genital ulceration, Raynaud phenomenon, sicca symptoms or peripheral stigmata suggestive of connective tissue disease.

Initial laboratory investigations demonstrated markedly elevated inflammatory markers without an obvious infective focus. He was admitted under the General Medicine team with a working diagnosis of fever of unknown origin and commenced empirically on intravenous piperacillin-tazobactam.

Despite broad-spectrum antimicrobial therapy, he continued to experience recurrent pyrexia and worsening constitutional symptoms. On the third day of admission, he developed photophobia and mild neck stiffness, raising concern for possible central nervous system infection. Following review by the microbiology team, antimicrobial therapy was escalated to intravenous ceftriaxone (2 g twice daily). Computed tomography (CT) of the head demonstrated no acute intracranial abnormality, and subsequent lumbar puncture revealed no significant cerebrospinal fluid abnormalities, making bacterial meningitis unlikely.

Serial laboratory investigations performed throughout the admission demonstrated persistently raised inflammatory markers with gradual improvement over time. Progressive thrombocytosis developed during admission, accompanied by mild normocytic anaemia and transient transaminitis. Renal function and electrolyte measurements remained stable throughout the admission (Table 1).

**Table 1:** Serial blood investigations Serial blood investigations showing progressive thrombocytosis, mild normocytic anaemia and transient transaminitis, with normal renal function and electrolytes. Hb: haemoglobin, WCC: white cell count, PLT: platelet, CRP: C-reactive protein, ESR: erythrocyte sedimentation rate, Na: sodium, K: potassium, eGFR: estimated glomerular filtration rate, ALT: alanine transaminase, CK: creatinine kinase

Name of test	Patient value					Normal range
	Day 1	Day 3	Day 9	Day 12	Day 16	
Hb	129 g/L	121 g/L	109 g/L	103 g/L	113 g/L	130-170 g/L
WCC	5.5 × 10^9^/L	5.1 × 10^9^/L	4.5 × 10^9^/L	5 × 10^9^/L	5 × 10^9^/L	4-10 × 10^9^/L
PLT	222 × 10^9^/L	274 × 10^9^/L	564 × 10^9^/L	570 × 10^9^/L	617 × 10^9^/L	150-450 × 10^9^/L
CRP	235 mg/L	151 mg/L	94 mg/L	76 mg/L	38 mg/L	<5 mg/L
ESR	105 mm/h	-	-	-	-	(0-50) 1-7 mm/h (male)
Urea	4.5 mmol/L	5.02 mmol/L	7 mmol/L	7 mmol/L	7 mmol/L	2.5-7.8 mmol/L
Creatinine	71 mmol/L	67 mmol/L	56 mmol/L	56 mmol/L	58 mmol/L	59-117 mmol/L
Na	134 mmol/L	136 mmol/L	135 mmol/L	134 mmol/L	135 mmol/L	133-146 mmol/L
K	4.1 mmol/L	4.3 mmol/L	4.3 mmol/L	4.7 mmol/L	4.8 mmol/L	3.5-8.3 mmol/L
eGFR	>90 g/L	>90 g/L	>90 g/L	>90 g/L	>90 g/L	0-21 g/L
Total protein	82 g/L	85 g/L	78 g/L	80 g/L	93 g/L	60-80 g/L
Globulin	48 g/L	55 g/L	50 g/L	52 g/L	60 g/L	20-35 g/L
Bilirubin	8 umol/L	6 umol/L	7 umol/L	7 umol/L	7 umol/L	0-21 umol/L
ALT	16 U/L	33 U/L	82 U/L	56 U/L	47 U/L	<50 U/L
CK	31 U/L	-	33 U/L	-	-	40-320 U/L
Magnesium	-	-	0.88 mmol/L	0.87 mmol/L	0.88 mmol/L	0.70-1.0 mmol/L
Calcium	-	2.36 mmol/L	2.37 mmol/L	2.04 mmol/L	2.05 mmol/L	2.20-2.60 mmol/L

Given the persistent fever, an extensive infectious disease workup was undertaken. Serological testing demonstrated positive Epstein-Barr virus (EBV) IgM with detectable EBV DNA, consistent with recent or reactivated EBV infection. Parvovirus B19 IgM and IgG were also positive (Table 2).

**Table 2:** Serological test results Serological test shows recent infection with evolving immunity. An elevated ASO titre indicated recent streptococcal exposure. EBV: Epstein-Barr virus, DNA: deoxyribonucleic acid, ASO: antistreptolysin O, PCR: polymerase chain reaction

Name of test	Patient value	Normal range
EBV DNA (qualitative PCR)	Detected-720 EBV DNA copies/mL	Negative
EBV IgM	Positive	Negative
Parvovirus IgM	Positive	Negative
Parvovirus IgG	Positive	Negative
ASO titre	400 IU/mL	<200 IU/mL

In contrast, extensive microbiological investigations were otherwise negative. Four sets of blood cultures and urine cultures showed no growth. Molecular testing was negative for bacterial 16S rRNA polymerase chain reaction (PCR), fungal 18S rRNA PCR, mycobacterial DNA, cytomegalovirus PCR, enterovirus PCR, and both *Leptospira* serology and PCR. Human immunodeficiency virus (HIV) serology and interferon-gamma release assay (QuantiFERON-TB Gold) were also negative (Table 3).

**Table 3:** Negative investigations rRNA: ribosomal ribonucleic acid, DNA: deoxyribonucleic acid, TB: tuberculosis, HIV: human immunodeficiency virus, PCR: polymerase chain reaction, CMV: cytomegalovirus

Name of test	Patient value
Four blood culture sets	No growth
Urine culture	No growth
Bacterial 16S rRNA PCR	Negative
Fungal DNA (18S RNA)	Negative
Mycobacterial DNA testing	Negative
QuantiFERON-TB Gold	Negative
HIV serology	Negative
*Leptospira* serology and PCR	Negative
CMV PCR	Negative
Enterovirus PCR	Negative

During the first week of admission, the patient’s clinical condition deteriorated further with severe pain affecting both upper and lower limbs, progressive proximal muscle weakness and increasing difficulty mobilising. There was no dysphagia or respiratory muscle involvement. In view of these evolving musculoskeletal features, rheumatology review was requested together with autoimmune serology and magnetic resonance imaging (MRI) of the thighs to investigate for inflammatory myopathy or systemic autoimmune disease.

**Figure 1:**
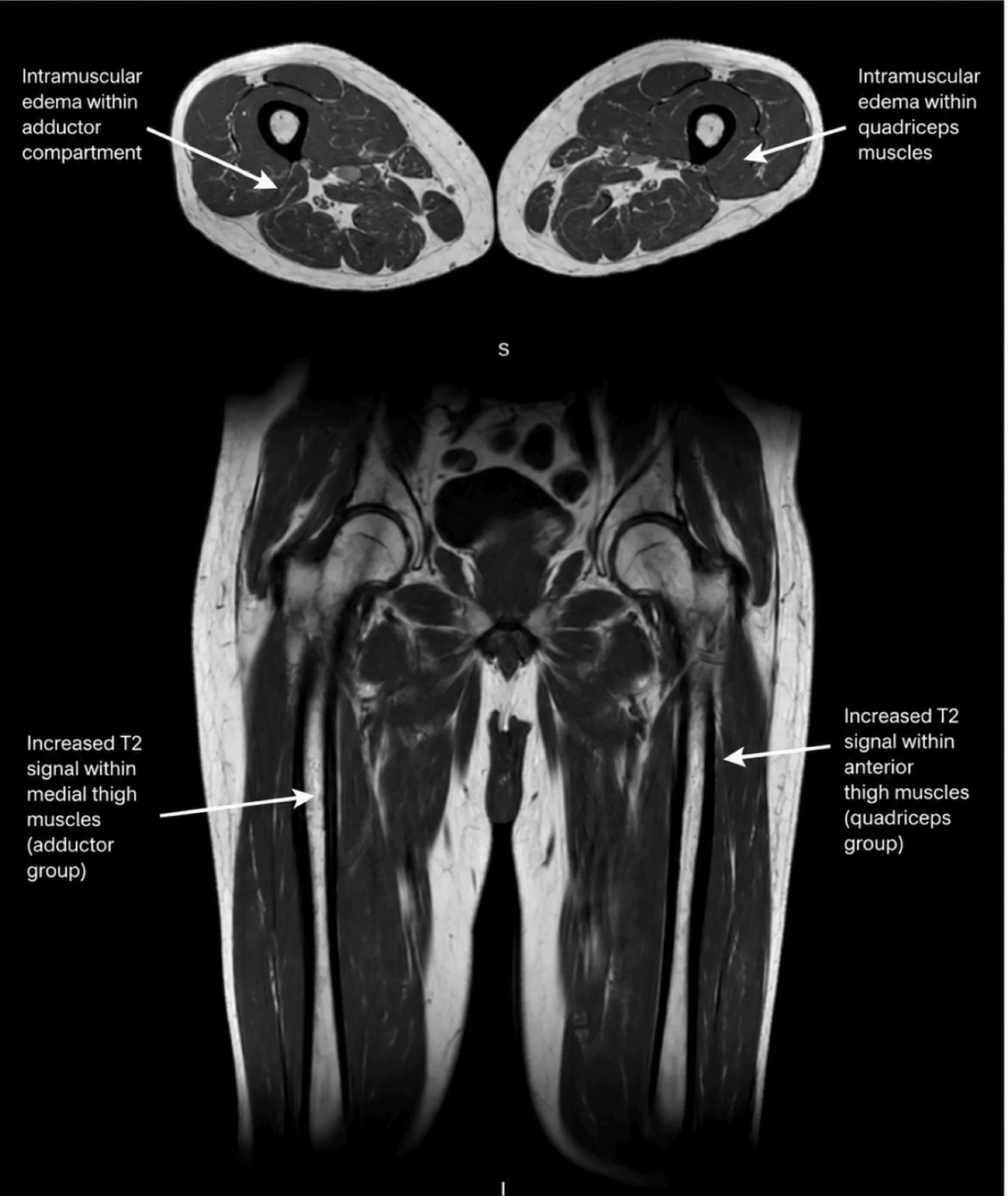
MRI of the thighs demonstrating bilateral posterior paraspinal muscle oedema with trace fluid tracking along the iliopsoas muscles (greater on the right) and small hip and knee joint effusions, consistent with inflammatory myositis and inflammatory arthritis MRI: magnetic resonance imaging

MRI demonstrated bilateral posterior paraspinal muscle oedema with trace fluid tracking along the iliopsoas muscles, more pronounced on the right, together with small bilateral hip and knee joint effusions. These appearances were compatible with inflammatory myositis and inflammatory arthritis (Figure 1).

The autoimmune screen revealed a strongly positive antinuclear antibody together with anti-Ro52, anti-Ro60 and anti-La antibodies consistent with primary Sjögren syndrome despite the absence of sicca symptoms. There was associated polyclonal hypergammaglobulinaemia with elevated IgG, IgA and IgM concentrations. Anti-double-stranded DNA antibodies, anti-RNP/Sm, anti-cyclic citrullinated peptide antibodies and antineutrophil cytoplasmic antibody (ANCA) were negative, making systemic lupus erythematosus, rheumatoid arthritis and ANCA-associated vasculitis less likely (Table 4).

**Table 4:** Immunological findings demonstrating positive ANA with strongly positive anti-Ro52, anti-Ro60 and anti-La antibodies associated with polyclonal hypergammaglobulinaemia ANA: antinuclear antibody, CTD: connective tissue diseases, dsDNA: anti-double-stranded deoxyribonucleic acid, SLE: systemic lupus erythematosus, Sm: Smith, anti-CCP: anti-cyclic citrullinated peptides, ANCA: anti-neutrophil cytoplasm antibodies, ENA: extractable nuclear antigen

Name of test	Patient value	Normal range
ANA (CTD screen)	Positive (29.4) ratio	0-0.69
dsDNA antibody	2.61 IU/mL	1-4 IU/mL
Ro52	Strong positive	Negative
Ro60 antibody	Strong positive	Negative
La antibody	Strong positive	Negative
RNL/Sm	Negative	Negative
Anti-CCP	2.3 U/mL	0-6.9 U/mL
MPO-ANCA	0.2 IU/mL	0-3.4 IU/mL
pANCa	0.8 IU/mL	0-1.9 IU/mL
ENA	>55 ratio	0-0.69
IgM	4.96 g/L	0.50-2.00
IgG	23.65 g/L	6-16 g/L
IgA	4.42 g/L	0.80-4.00 g/L
Kappa light chain	111.09 mg/L	3.30-19.40 mg/L
Lambda light chain	129.89 mg/L	5.71-26.30 mg/L
Kappa/lambda ratio	0.86	0.26-1.65
Anti-cardiolipin antibody	2.5 gplu/mL	0-9.9 gplu/mL
Complement C3	1.4 g/L	0.9-1.8 g/L
Complement C4	0.12 g/L	0.14-0.54 g/L
Liver antibody profile	Negative	Negative

**Figure 2:**
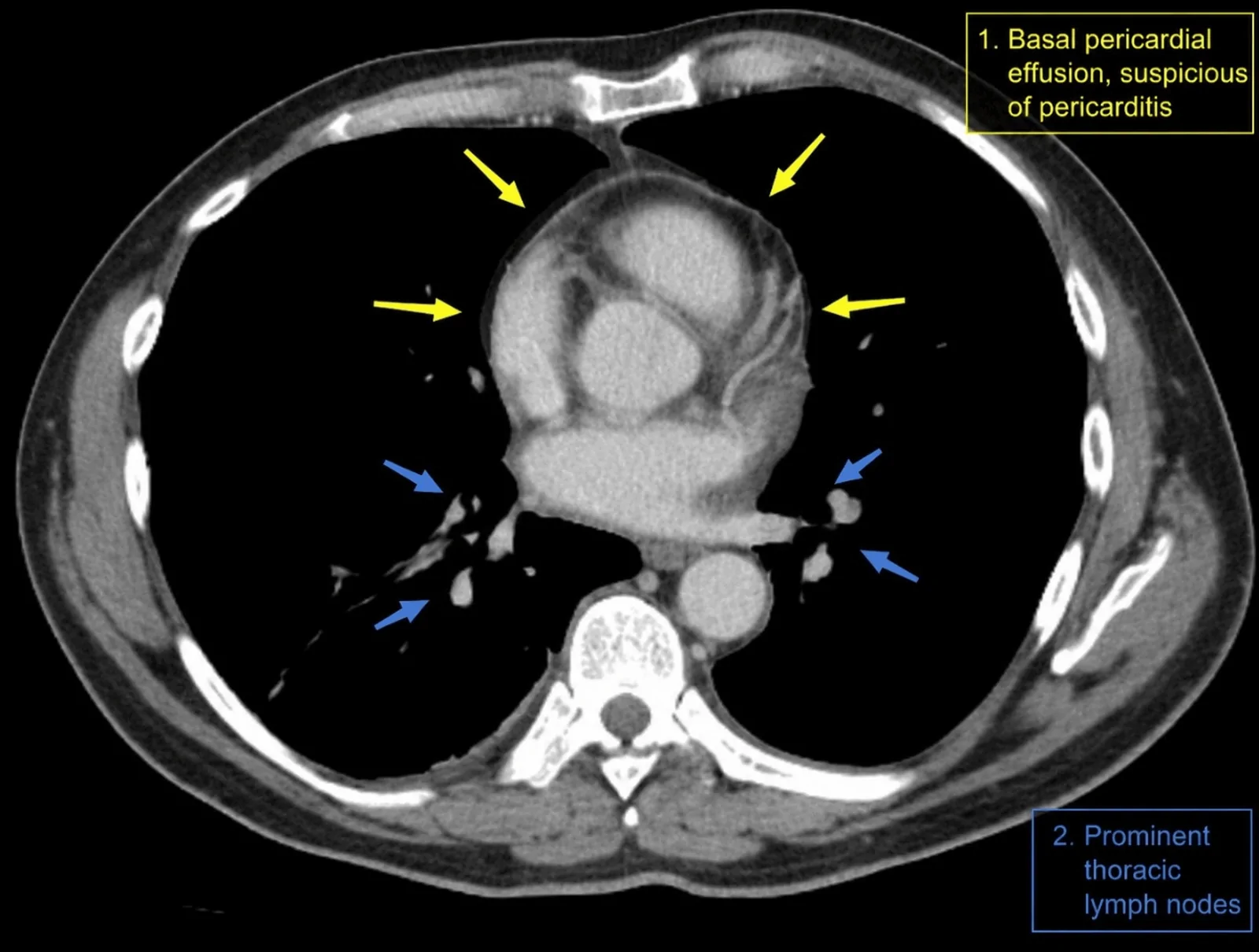
CT of the chest, abdomen and pelvis demonstrating pericardial effusion up to 1.3 cm with pericardial enhancement and thoracic lymphadenopathy CT: computed tomography

As the patient remained febrile with persistent systemic inflammation, CT of the chest, abdomen and pelvis was performed to investigate for occult infection, malignancy or other significant pathology. Imaging demonstrated a pericardial effusion measuring up to 1.3 cm with associated pericardial enhancement consistent with pericarditis, together with multiple enlarged thoracic lymph nodes (Figure 2).

Subsequent cardiac MRI demonstrated normal biventricular size and preserved systolic function with mild septal bounce and inspiratory septal flattening, indicating ventricular interdependence. Global pericardial enhancement with associated oedema and a small area of basal inferolateral mid-wall enhancement were present, together with mild myocardial oedema. No myocardial infarction or intracardiac vegetation was identified. These findings confirmed acute myopericarditis.

At this stage, the differential diagnosis remained broad and included occult bacterial infection, viral infection, tuberculosis, connective tissue disease, adult-onset Still’s disease, inflammatory myopathy, systemic vasculitis, sarcoidosis, Hodgkin lymphoma and non-Hodgkin lymphoma.

**Figure 3:**
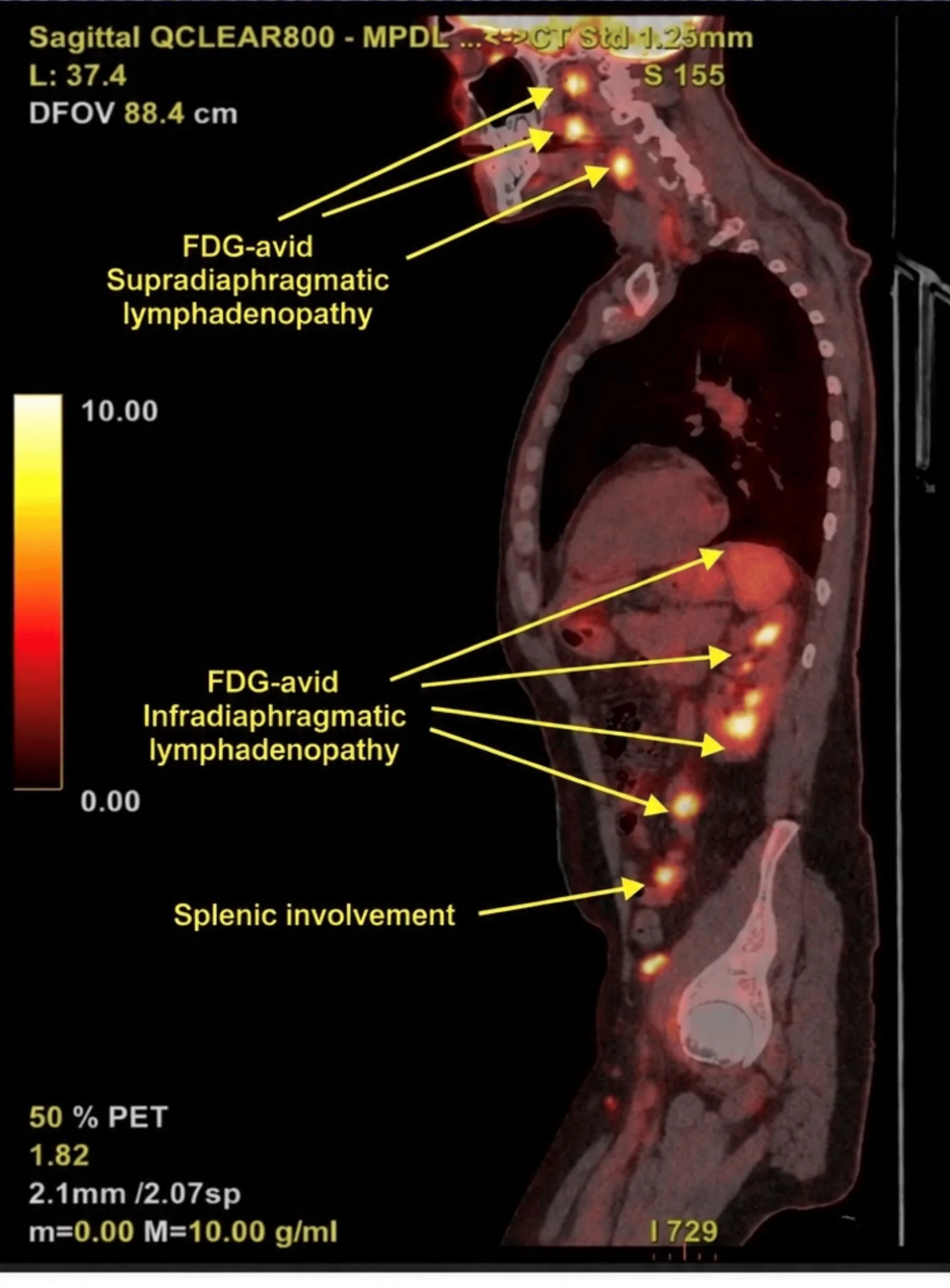
FDG PET-CT demonstrating metabolically active supra- and infradiaphragmatic lymphadenopathy with splenic uptake suggestive of lymphoproliferative disease PET: positron emission tomography, CT: computed tomography, FDG: fluorodeoxyglucose

As no unifying diagnosis had been established despite extensive investigations, fluorodeoxyglucose positron emission tomography (FDG PET-CT) was performed to evaluate for occult inflammatory or malignant disease. PET-CT demonstrated FDG-avid supra- and infradiaphragmatic lymphadenopathy with splenic involvement, raising significant concern for lymphoma (Figure 3).

To establish a definitive diagnosis, a single-site excisional cervical lymph node biopsy was performed. Histopathological examination demonstrated reactive lymphoid hyperplasia with no evidence of Hodgkin lymphoma, non-Hodgkin lymphoma, metastatic malignancy or granulomatous inflammation, effectively excluding an underlying lymphoproliferative disorder.

The patient initially received empirical intravenous piperacillin-tazobactam for three days before antimicrobial therapy was changed to ceftriaxone following the development of possible meningeal symptoms. Symptomatic treatment consisted of regular paracetamol and intermittent ibuprofen. No corticosteroids, colchicine, intravenous immunoglobulin or immunosuppressive therapy were administered.

Remarkably, spontaneous clinical improvement occurred during the second week of admission. Fever frequency progressively reduced, muscle strength improved and inflammatory markers continued to decline. By discharge during the third week of admission, the CRP had decreased from 235 mg/L to 38 mg/L, although thrombocytosis persisted. The patient was clinically well and discharged with outpatient follow-up in haematology, rheumatology and cardiology.

Following multidisciplinary review, the haematology team concluded that the lymphadenopathy, splenomegaly and immunoglobulin abnormalities represented a reactive inflammatory process rather than lymphoma. Owing to the patient’s spontaneous recovery, rheumatology cancelled the planned muscle biopsy.

At two-month follow-up, the patient remained clinically well with no recurrence of fever or progressive systemic symptoms. Repeat imaging had not yet been performed.

## Discussion

This case illustrates acute EBV infection presenting as a systemic inflammatory syndrome mimicking both lymphoma and connective tissue disease. This represents a rare manifestation of acute EBV infection and highlights the diagnostic challenges posed by its atypical presentation. Initial concern for lymphoma was driven by constitutional symptoms, FDG-avid generalized lymphadenopathy [2], splenic involvement and elevated inflammatory markers. Polyclonal hypergammaglobulinaemia further increased diagnostic uncertainty. Histology confirmed reactive lymphadenopathy, excluding malignancy. The autoimmune serology profile, including ANA, Ro52, Ro60 and La positivity with low complement levels, suggested a connective tissue disease. However, the absence of clinical criteria and spontaneous resolution without immunosuppression argued against a defined autoimmune disorder.

EBV-related immune dysregulation may explain transient autoantibody positivity through polyclonal B-cell activation and molecular mimicry [6]. This mechanism can produce serological patterns that mimic systemic autoimmune disease.

Cardiac involvement in EBV infection is uncommon. Myopericarditis has been described as a rare manifestation of EBV infection [3,7]. In this case, diagnosis was established only on cardiac MRI, which is recognized as the most sensitive modality for non-ischaemic myocardial inflammation [8]. Similar cases of EBV-associated myopericarditis have been reported in immunocompetent individuals [9,10]. The combination of EBV IgM positivity, detectable EBV DNA, reactive lymphadenopathy, polyclonal hypergammaglobulinaemia and spontaneous clinical recovery all supports EBV-associated systemic immune activation as the unifying diagnosis [3,6]. Although parvovirus B19 serology was also positive, the overall pattern of generalized lymphadenopathy and immune activation is more consistent with EBV infection [2,3]. The patient demonstrated spontaneous clinical improvement approximately one week after admission without immunosuppressive therapy. Such a course is atypical for lymphoma or systemic autoimmune disease and supports a self-limiting viral inflammatory process [3,6]. Recent reports describe isolated autoimmune or cardiac manifestations of EBV infection [9,10]. However, the simultaneous presence of PUO, generalized FDG-avid lymphadenopathy, transient autoantibody positivity, polyclonal hypergammaglobulinaemia and MRI-confirmed myopericarditis appears rare. Long-term follow-up is warranted, as EBV-associated immune dysregulation may occasionally precede defined autoimmune disease [6].

## Conclusions

This case highlights the ability of acute EBV-associated immune activation to mimic lymphoma and systemic autoimmune disease. Histological confirmation of PET-avid lymphadenopathy proved essential in excluding malignancy. Clinicians should consider EBV-associated inflammatory syndromes in patients presenting with PUO, generalized lymphadenopathy, positive autoimmune serology and myopericarditis, particularly after exclusion of more common infectious, autoimmune and malignant causes.
